# Items

**Published:** 1881-11

**Authors:** 


### Items.

Cariatides from Cadavers. — An engineer of Havana, named Crulz, proposes the following way of embalming. The body is placed in a bath composed of equal parts of lime and clay, dissolved in a sufficient quantity of water, then it is covered with another layer of natural cement, destined to absorb the excess of water, after which the cadaver is submerged in a bath of pitch, and covered finally with a layer of lime; the contact, only, between the lime and calcareous cement being sufficient to solidify the pitch rapidly; a thick coating being formed in this manner, which possesses the same properties as the pitch of Judea, a substance to which the Egyptian mummies owe their peculiar indestructibility. As can be readily understood, a subject so prepared can exhale no marked odor; the different layers of lime, clay and pitch forming around it a kind of solid wrapping, which is opposed effectually to the disengagement of gases. A cadaver, after being treated in this manner, is deposited in the interior of a mould, which is filled with the following mixture, that very soon solidifies, and is transformed into stone: Cement, five parts; sand, three parts; ashes, two parts; water, a sufficiency. The stones which are obtained by this process acquire a remarkable solidity. Obituary inscriptions can be engraved upon them; they can be placed in mausolea, or may serve for the construction of sepulchral monuments of various forms: (Boston Med. and Surg. Journal.) We would suggest silicate of potassium (liquid glass) as a coat, which would be highly artistic, and through this process lawns and gardens could be adorned with the well-preserved persons of ancestors, in place of common statues. Or, if preferred, the busts of grandparents could be in the same manner preserved and utilized as mantel ornaments, which would enhance the veneration of the departed.

STATE MEDICAL SOCIETY JOURNALS. — Under the title of *Ohio Medical Journal*, the *Ohio Medical Recorder* has become the journal of the Ohio State Medical Society, and will in future take the place of the usual volume of transactions. The editor in charge is the secretary of the society, and was formerly managing editor of the *Recorder* ; he is to be assisted by four associate editors from as many of the larger cities in different parts of the State. We shall watch over the career of our contemporary with special attention. Although long suggested, this is the first experiment of the kind in this country. The disadvantages are numerous, if they do not outweigh the advantages of such an arrangement. There is certain discredit thrown on medical societies by those who prefer not to belong to any, and they are numerous, while they all take at least one medical paper. The editorial department soon weakens, for one reason or another, or may not exist at all, as with the *British Medical Journal.* Cosmopolitan summary of medical science and progress, which is just what the majority of readers need, finds nobody to attend to it. At the same time, a large amount of trash and useless material fill up the pages of the periodical. In journals, as in other literary undertakings, individual efforts generally succeed the best.

LEPROSY IN LOUISIANA.—The annual report of the Louisiana Board of Health for 1880, just issued, contains a detailed statement of the progress of Asiatic leprosy in that State during the last century. It was brought in 1680 to the West Indies, by the negro slaves, and thence to Louisiana. In 1778 this disease was so prevalent among the blacks, together with the African elephantiasis, and another equally horrible, named yaws, peculiar to Guinea negroes, that a hospital for lepers was established in New Orleans. At the present time, the majority of lepers in that city are found to be whites, of French, German and Russian extraction. Although the disease is infectious, New Orleans, it appears, has no separate asylum for these incurable patients, and they are received into the Charity Hospital, and placed in the crowded wards, to scatter death: (Medical and Surgical Reporter.) Another nidus of Asiatic leprosy exists near Shediac, New Brunswick, where an asylum has been in existence ever since the French domination. It seems that the first cases of leprosy came to that place from Norway.

A SERIES OF PRIZE QUESTIONS.—The prize questions which are proposed by the various medical bodies throughout the world may be taken to indicate the points on which information is most desired. The Royal Academy of Brussels announces a prize to be awarded in January, 1884, of $1,600 for the best explanation of the pathology and therapeutics of the diseases of the nerve centers, especially epilepsy, illustrated by clinical data and experiments. If the essayist discovers a successful treatment for epilepsy, he is to receive in addition $5,000.

Other prizes offered by the Academy are for essays on the questions:

"What is the nature of the influence of innervation on the nutrition of tissues?"

"What part do living germs play in the etiology of diseases?"

"What effect does drying, as a means of preservation of plants used in medicine, exercise on their medicinal properties?"

Still another prize ($240), especially designated as for *international* concurrence, is offered by the same body for the best dissertation on the subject—"The value of spectral analysis for forensic medicine and sanitary science, exhibited by original experiments and investigations."

The Academy of Medicine of Turin, Italy, offers a prize of $4,000 for the best essay "On the physio-pathology of the blood." It is open to the world, but must be either in the Italian, French or Latin tongue.

In Paris, the Société de Chirurgie has lately announced three prizes for competition this year. "The subjects are: "The remote results of ovariotomy;" "The etiological rôle of contusions in the development of neoplasms." "The history and theory of union by the first intention."

The American Neurological Association offers a prize of $500, to be known as the "William A Hammond Prize," and to be awarded at the meeting in June, 1882, to the author of the best essay on the "Functions of the thalamus in man;" and the Warren prize committee offer a premium of $400 for the best essay on "Chronic Bright's disease."

Following are the prizes proposed by the French Academy of Medicine for 1882:

Academy Prize.—Generalized arterial atheroma, and its influon the nutrition of the organs,—$200.

Baron Portal Prize.—The lymphatics under a pathological point of view,—$400.

Mme. B. de Civrieux Prize.—Researches respecting the causes of locomotor ataxia,—$400.

Dr. Capuron Prize.—The lochia in a normal and in a pathological state,—$400.

Dr. Godard Prize.—The best work on internal pathology,—$300.

Dr. Desportes Prize.—The best work on practical medical therapeutics,—$400.

Mme. H. Buignet Prize.—The best work on the application of physics or chemistry to the medical sciences,—$300.

Dr. Orfila Prize.—Veratrine, sabadilline, black hellebore and veratrum album,—$800.

Dr. Itard Prize.—Best work on practice,—$600.

Mr. and Mrs. St. Paul Prize.—$5,000 for a specific for diphtheria.—*Bulletin de l'Académie de Médicine.*

NOTES ON ANTHROPOLOGICAL STUDIES.—The question of the size and height of men receives a careful study in the *Verhandlungen* from Herr Ranke and others. It has been supposed by some that the earlier races were larger in size, by others that they were smaller than the people now occupying the same territory. Herr Ranke demonstrates, from a large number of measurements, that man is a creature of the soil on which he lives (?); that certain localities produce on an average men of greater stature than other localities, and that as a general rule, elevations above the sea level and mountainous regions are peopled by the larger races. No diminution in stature, nor any increase, can be proved to have taken place since even pre-historic times in Europe. The skeletons from the oldest tumuli occasionally show individuals to have reached the astonishing height of two meters (6 ft. 6 in.), but as a rule are not in excess of the stature of the population now dwelling on the same soil.

A REMARKABLE MODE OF ZOÖLOGICAL DISTRIBUTION.—M. Méguin lately presented to the Société de Biologie a collection of fifteen leeches, of a species foreign to those found in France. They were adhering to the walls of the mouths of horses just returned from the Tunis campaign. They had been camping near Bizerte. All the brooks of Northern Africa contain that kind of leeches, called *hæmopis sanguisuga*, whose jaws are too weak to bite the skin, so that they enter into the animals' mouths when these come to drink, and cling to the mucous membrane. They may live in this situation for weeks, causing hæmorrhages, and even asphyxia, if they enter the larynx. But they die almost immediately when introduced into the stomach or the rectum.

AT the meeting of the Societé d' Anthropologie, held June 2, 1881, Dr. Magistos exhibited an hermaphrodite, born in 1841, and christened Ernestine Guériau. About the thirteenth year there had been some slight menstruation followed by enlargement of the breasts. When seventeen and a half years old this individual married a man, but the sexual intercourse was not quite satisfactory as it had a pseudo-vagina not very deep, and a penis susceptible of incomplete erection. Husband died in 1871. After this the hermaphrodite had intercourse with women, and owns that it had them before the husband's death also. Height 1 m. 78; black hair, shaves every other day; form is masculine though the voice is feminine; breasts are covered with hair. The pubis is covered with hair, and so is the perineum. There are two labia continuous above with a penis the size of a child's, 4 or 5 c; the glans is imperforate, and there is no prepuce. It is curved with concavity downward when excited. The left labium is larger and contains a testicle of normal size, the left contains a smaller one. At the base and inferior part of the penis is the meatus urinarius whence flow the urine and the sperm.

